# The Influence of Age and Comorbidities on Telehealth Usability Perceptions in Patients with Hypertension: A Cross-Sectional Study in Saudi Arabia

**DOI:** 10.3390/healthcare14203409

**Published:** 2026-10-13

**Authors:** Hekmat Yousef Al-Akash, Murad Sawalha, Anwar Jabr Nawi Alshammari, Hawazen Rawas, Mohannad Eid AbuRuz, Zyad Saleh, Osama Al-Kouri, Nabeel Al-Yateem, Alaa Mohammad Ahmed, Ahmad Rajeh Saifan

**Affiliations:** 1 Clinical Nursing Department, Faculty of Nursing, Applied Science Private University (ASU), Amman 11937, Jordan; 2 Department of Maternal, Child and Family Health Nursing, Faculty of Nursing, The Hashemite University, Zarqa 13133, Jordan; msawalha@hu.edu.jo; 3 Faculty of Nursing, Applied Science Private University (ASU), Amman 11937, Jordan; anwrjn1997@gmail.com; 4 Nursing Department, College of Medicine and Health Sciences, Arabian Gulf University, Manama P.O. Box 26671, Bahrain; hawazenor@agu.edu.bh; 5 Basic Nursing Department, Nursing College, Yarmouk University, Irbid 21163, Jordan; mohannad.aburuz@yu.edu.jo (M.E.A.); o.alkouri@yu.edu.jo (O.A.-K.); ahmad.saifan@yu.edu.jo (A.R.S.); 6 Department of Clinical Nursing, School of Nursing, The University of Jordan, Amman 11942, Jordan; zyad.saleh@ju.edu.jo; 7 Department of Nursing, College of Health Sciences, University of Sharjah, Sharjah P.O. Box 32223, United Arab Emirates; nalyateem@sharjah.ac.ae; 8 Sheikh Shakhbout Medical City (SSMC), Abu Dhabi 11001, United Arab Emirates; almoahmed@ssmc.ae

**Keywords:** telehealth, hypertension, usability, age, comorbidity, patient-reported outcomes

## Abstract

**Highlights:**

**What are the main findings?**

- Among 266 patients with hypertension in Saudi Arabia, perceived telehealth usability was 93.11 ± 21.58 on the 21-item TUQ, corresponding to a mean item score of 4.43 ± 1.03.
- Perceived telehealth usability differed significantly across age groups, and age in years was inversely associated with the TUQ score in the corrected multivariable analysis (B = −0.357, 95% CI −0.525 to −0.190; *p* < 0.001).
- Usability varied substantially across specific comorbidity categories. Compared with participants with hypertension only/no additional comorbidity, participants in the broad “other comorbidities” category had higher adjusted TUQ scores (B = 23.292, 95% CI 12.392–34.192; *p* < 0.001), whereas diabetes, chronic kidney disease, and respiratory conditions were not independently associated with the TUQ score.

**What are the implications of the main findings?**

- Telehealth services for hypertension may benefit from age-sensitive design features, such as simplified navigation, clear visual presentation, and alternative communication options, particularly for patients who experience greater usability barriers.
- Healthcare providers and policymakers should avoid assuming that all patients with comorbidities have similar telehealth experiences. Monitoring usability across specific clinical subgroups may help identify patients who require additional technical or patient-centered support.

**Abstract:**

**Background/Objectives:** Telehealth has promising opportunities for hypertension management through remote monitoring and enhanced patient–provider communication. However, the usability of these services may vary across patient populations, particularly with respect to age and clinical complexity. This study examined the association of age and specific comorbidity categories with perceived telehealth usability among patients with hypertension in Saudi Arabia. **Methods:** An analytical cross-sectional study using questionnaire data linked with retrospective electronic health-record information was conducted among adults with physician-diagnosed hypertension who received telehealth follow-up following hospital discharge. Participants completed the Arabic version of the Telehealth Usability Questionnaire (TUQ). Sociodemographic and clinical data were extracted from electronic health records. Age in years was entered as a continuous variable in the primary multivariable model, while age group was examined as a categorical sensitivity analysis. Comorbidity was entered as a nominal categorical variable using hypertension only/no additional comorbidity as the reference group. Because group variances were heterogeneous, Welch’s ANOVA and Games–Howell post-hoc comparisons were used for group comparisons. **Results:** We included 266 adults with physician-diagnosed hypertension. Mean age among the 265 participants with valid age entries was 48.38 years (SD 18.80), and 56.0% were female. The mean total TUQ score was 93.11 ± 21.58, corresponding to a mean item score of 4.43 ± 1.03. According to the prespecified mean-item scoring framework, this represented moderate perceived usability. Significant differences were observed across age groups (Welch’s F(5,46.07) = 8.96, *p* < 0.001; η^2^ ≈ 0.177), with the highest mean TUQ score among participants aged 20–30 years (110.75 ± 31.72) and the lowest among those aged >70 years (82.53 ± 7.33). In the corrected multivariable model, age was inversely associated with the TUQ score (B = −0.357, 95% CI −0.525 to −0.190; *p* < 0.001; β = −0.314). Compared with hypertension only/no additional comorbidity, the “other comorbidities” group had higher TUQ scores (B = 23.292, 95% CI 12.392–34.192; *p* < 0.001), whereas diabetes, chronic kidney disease, and respiratory conditions were not independently associated with the TUQ score. **Conclusions:** Perceived telehealth usability among patients with hypertension varied according to age and specific clinical characteristics. Older age was associated with less favorable usability perceptions, whereas the pattern across comorbidity categories was heterogeneous rather than uniformly positive or negative. These findings support age-sensitive, patient-centered telehealth design and targeted usability support for clinical subgroups experiencing greater barriers to digital care.

## 1. Introduction

The rapid expansion of telehealth has created new opportunities to support the long-term management of chronic diseases, including hypertension [1]. Telehealth can facilitate communication with healthcare professionals, improve access to follow-up, and support remote monitoring and self-management, but the benefits of these services depend not only on their availability but also on whether patients perceive them as usable and relevant to their needs [2]. A systematic review found significant positive associations between perceived ease of use and intention to use healthcare technology, perceived usefulness and intention to use, and social influence and intention to use [3].

Hypertension is suitable for telehealth because disease management requires repeated assessment, ongoing treatment adjustment, and sustained patient participation, all of which can be supported through digital communication and remote monitoring [4]. Recent evidence indicates that structured remote-care models can lead to meaningful benefits. In this regard, a mixed-methods study of patients enrolled in a remote patient-monitoring program found that highly engaged participants were older than those with lower engagement and had substantially greater odds of achieving normotension, demonstrating that older age does not preclude successful participation in digital hypertension care when appropriate support is available [5].

The usability of telehealth may differ considerably between patient groups, particularly according to age and clinical complexity. Older adults may encounter greater difficulties with digital interfaces, physical or cognitive demands, and technology-related confidence, although these barriers are modifiable through appropriate design and support [6]. Limited digital literacy, physical and cognitive limitations, infrastructural deficiencies, usability problems, privacy concerns, and mistrust were identified as recurrent barriers to digital-health adoption [7]. Moreover, perceived usefulness was the strongest determinant of digital-health adoption among older adults, while perceived ease of use was strongly associated with perceived usefulness, and the strength of these relationships varied by culture, age group, and technology type [8].

Despite this growing evidence, existing studies have examined technology acceptance, satisfaction, engagement, or blood-pressure control rather than multidimensional patient-reported usability, and many have focused on older adults or specific digital interventions rather than people with hypertension across a broad age range [9]. Furthermore, patients with multiple chronic conditions may have distinctive patterns of telehealth use and experience, yet the contribution of specific comorbidity categories to perceived usability remains not well characterized [10]. Therefore, we aimed to examine the association of age and specific comorbidity categories with perceived telehealth usability among patients with hypertension in Saudi Arabia.

## 2. Materials and Methods

### 2.1. Design

An analytical cross-sectional study using questionnaire data linked with retrospective electronic health-record information was conducted to examine the association of age and clinical characteristics, including specific comorbidity categories, with patients’ perceptions of telehealth usability. The analysis was based on data collected from adults with physician-diagnosed hypertension who had received telehealth follow-up after hospital discharge. The study was conducted in two large hospitals in Sakaka, Al-Jouf Region, Saudi Arabia. The participating facilities included general medical and chronic disease management services where patients with hypertension were routinely admitted and subsequently followed through telehealth services.

Ethical approval was obtained from the university and participating hospital ethics committees. Participants provided informed consent before completing the questionnaire. Patient identifiers were removed prior to analysis, and de-identified information was restricted to authorized study personnel.

### 2.2. Study Population

The study population consisted of adults with hypertension who had used telehealth services following hospital discharge. Participants were eligible if they were aged 18 years or older, had a documented diagnosis of hypertension, had been discharged from hospital within the preceding 12 months, had received telehealth follow-up within 30 days of discharge, and had complete clinical information and telehealth usability responses for analysis. A purposive sampling strategy was used.

### 2.3. Measures and Variables

Sociodemographic and clinical information was obtained using a structured data-collection form and information extracted from electronic health records. Variables included age, sex, hypertension severity at admission, comorbid conditions, and 30-day readmission status. Hypertension severity was classified according to the 2017 American Heart Association/American College of Cardiology framework. Stage I hypertension was defined as systolic blood pressure (SBP) 130–139 mmHg and/or diastolic blood pressure (DBP) 80–89 mmHg; Stage II hypertension was defined as SBP ≥ 140 mmHg and/or DBP ≥ 90 mmHg; and the category designated “hypertensive crisis” in the study instrument was defined as SBP > 180 mmHg and/or DBP > 120 mmHg. Classification was based on disease severity at admission as recorded in the electronic health record. The term “hypertensive crisis” was used as a blood-pressure-based operational category and did not require evidence of acute target-organ damage; therefore, it should not be interpreted as synonymous with hypertensive emergency.

The comorbidity variable contained one response category per participant. The observed categories were hypertension only/no additional comorbidity, diabetes, chronic kidney disease, respiratory conditions, and other comorbidities. For the multivariable analysis, these categories were treated as nominal categorical predictors using indicator variables, with hypertension only/no additional comorbidity as the reference category.

### 2.4. Telehealth Service

Eligible participants had received post-discharge telehealth follow-up within 30 days of hospital discharge. The research questionnaire was administered electronically using Google Forms. Google Forms was used only for questionnaire/data collection and was not itself considered the telehealth intervention. The clinical telehealth contact was telephone-based.

### 2.5. Telehealth Usability Questionnaire

The primary outcome was perceived telehealth usability, assessed using the Arabic version of the Telehealth Usability Questionnaire (TUQ) [11]. The TUQ is a 21-item instrument consisting of six domains: usefulness, ease of use and learnability, interface quality, interaction quality, reliability, and satisfaction and future use. In the present questionnaire/data structure, these domains comprised 3, 4, 3, 4, 4, and 3 items, respectively. Each item is rated on a seven-point Likert scale ranging from 1 (strongly disagree) to 7 (strongly agree). The total score ranges from 21 to 147, with higher scores indicating more favorable perceptions of telehealth usability. The overall TUQ score was calculated by summing the 21 item responses. A mean item score was also obtained by dividing the total score by the number of items.

The Arabic TUQ has undergone cross-cultural adaptation and psychometric evaluation in Saudi Arabia [12]. In the present sample, internal consistency was assessed using Cronbach’s alpha. The total TUQ demonstrated α = 0.866, while domain-specific alpha coefficients ranged from 0.342 to 0.486. Because the total scale demonstrated substantially stronger internal consistency than the individual short domains, the total TUQ score was used as the primary inferential outcome and domain scores were interpreted descriptively.

### 2.6. TUQ Scoring

Each item of the TUQ is rated on a 7-point Likert scale. The total score is obtained by summing all 21 item scores, with a possible range of 21–147. Higher scores indicate more favorable usability perceptions [11,12].

For descriptive interpretation in the present study, the mean item score was categorized as low (≤3.0), moderate (3.1–5.0), or high (≥5.1) [13]. The overall mean item score of 4.43 ± 1.03 therefore falls within the moderate category.

### 2.7. Data Collection Procedure

Following institutional approval, eligible participants were contacted and invited to complete an electronic questionnaire. The electronic form contained study information and an informed-consent statement before the questionnaire items. Participants who agreed to participate completed the sociodemographic and clinical questions and the TUQ. Clinical information was obtained from electronic health records, and identifying information was removed before the analytical dataset was created.

Data-quality checks were performed before analysis. One participant had a non-numeric age entry and was excluded from age-specific analyses, leaving 265 valid age observations. A separate participant had a missing 30-day readmission response; this observation was excluded from complete-case multivariable regression, resulting in 264 observations for the corrected regression model. No values were imputed.

### 2.8. Statistical Analysis

Data were analyzed using IBM SPSS Statistics version 28. Continuous variables were summarized using means and standard deviations, whereas categorical variables were summarized using frequencies and percentages. Statistical significance was assessed using a two-sided significance threshold of *p* < 0.05.

The distribution of perceived telehealth usability was first summarized using the overall TUQ score and its six domain scores. Differences in mean TUQ scores across age groups, hypertension-severity categories, and comorbidity categories were examined using one-way analysis of variance (ANOVA). Because the assumptions of conventional ANOVA were not uniformly satisfied, the inferential group comparisons were based on Welch’s ANOVA when the homogeneity-of-variance assumption was violated. Within-group normality was assessed using the Shapiro–Wilk test and homogeneity of variances using Levene’s test. Following significant omnibus tests, Games–Howell post-hoc comparisons were used because they do not require equal variances. Omnibus effect sizes were summarized using η^2^, and pairwise differences were summarized using Hedges’ g. Comparisons between males and females were performed using an independent-samples t test, with Welch’s correction where appropriate.

Multiple linear regression was subsequently performed with total TUQ score as the dependent variable. Age in years was entered as a continuous variable in the primary model. Age group was entered as a categorical sensitivity analysis. Sex, hypertension severity, comorbidity category, and 30-day readmission status were entered as categorical predictors using indicator variables. Regression coefficients were reported as unstandardized coefficients (B), standardized coefficients (β), HC3-robust 95% confidence intervals (CIs), t statistics, and *p* values. Model-level information included R^2^, adjusted R^2^, and the overall robust Wald F statistic. Multicollinearity was assessed using variance inflation factors, and residual normality, homoscedasticity, and influential observations were examined using Shapiro–Wilk, Breusch–Pagan, and Cook’s-distance diagnostics, respectively. Because heteroscedasticity was detected, HC3 heteroscedasticity-robust standard errors were used for inference.

## 3. Results

### 3.1. Participant Characteristics

The study included 266 participants. Valid age information was available for 265 participants, with age ranging from 18 to 98 years and a mean of 48.38 (SD 18.80). The median valid age was 46 years. The largest age group was 51–70 years (*n* = 73, 27.4%), followed by participants aged 31–40 years (*n* = 57, 21.4%) and 41–50 years (*n* = 57, 21.4%). Women represented 56.0% of the sample (*n* = 149). Also, 105 participants (39.5%) had stage I hypertension, 98 (36.8%) had stage II hypertension, and 63 (23.7%) were classified as having a hypertensive crisis. Diabetes was the most frequently recorded comorbidity (*n* = 77, 28.9%). The comorbidity variable showed that 79 participants (29.7%) had hypertension only/no additional comorbidity, 77 (28.9%) had diabetes, 34 (12.8%) had chronic kidney disease, 32 (12.0%) had respiratory conditions, and 44 (16.5%) had other comorbidities. Among the 265 records with a valid readmission response, 152 participants (57.1%) were readmitted within 30 days and 113 (42.5%) were not; one record (0.4%) had a missing readmission response (Table 1).

### 3.2. Overall Telehealth Usability

Overall perceived telehealth usability was moderate according to the prespecified mean-item scoring framework. The mean total TUQ score was 93.11 (SD 21.58), with a median of 87.0 and a range of 41.0–147.0. The six usability domains showed similar ratings, although satisfaction and future use had the highest mean item score (4.54 ± 1.25), followed by interaction quality (4.48 ± 1.17) and reliability (4.47 ± 1.14). Usefulness recorded the lowest mean item score (4.32 ± 1.32). Cronbach’s alpha for the total TUQ was 0.866. Domain-specific alpha coefficients were 0.406 for usefulness, 0.384 for ease of use and learnability, 0.486 for interface quality, 0.368 for interaction quality, 0.388 for reliability, and 0.342 for satisfaction and future use (Table 2).

### 3.3. Variation in Telehealth Usability According to Age

Perceived telehealth usability differed significantly across age groups. Because Levene’s test indicated heterogeneity of variance (W = 13.71, *p* < 0.001) and several age groups showed significant departures from normality on the Shapiro–Wilk test, Welch’s ANOVA was used for the primary inference.

The mean TUQ score was highest among participants aged 20–30 years (110.75 ± 31.72) and lowest among those aged >70 years (82.53 ± 7.33).

Welch’s ANOVA demonstrated a significant overall difference across age groups (F(5,46.07) = 8.96, *p* < 0.001), with η^2^ ≈ 0.177. Games–Howell post-hoc comparisons showed that the 20–30-year group had higher TUQ scores than the 41–50-year group (adjusted *p* < 0.001), 51–70-year group (adjusted *p* = 0.001), and >70-year group (adjusted *p* < 0.001). The 31–40-year group had higher scores than the 41–50-year group (adjusted *p* = 0.040) and >70-year group (adjusted *p* = 0.001). The <20-year group also differed from the 20–30-year group (adjusted *p* = 0.001).

The observed group means did not decrease strictly monotonically across every age category. Accordingly, the results are interpreted as significant between-group differences and an overall inverse association with age rather than as evidence of a uniform decline at every successive age interval (Table 3, Table 4 and Table 5).

### 3.4. Telehealth Usability According to Sex and Hypertension Severity

Mean TUQ scores were higher among men than women (94.74 ± 23.65 vs. 91.82 ± 19.79), but it was not statistically significant (Welch’s *p* = 0.284).

Perceived usability also varied according to hypertension severity. Participants with stage I hypertension had the highest mean TUQ score (96.59 ± 24.63), followed by stage II hypertension (92.47 ± 20.22), while those classified as having hypertensive crisis had the lowest score (88.29 ± 16.98).

The conventional one-way ANOVA for hypertension severity produced F = 3.03, *p* = 0.050; Levene’s test was not significant (W = 2.72, *p* = 0.067), supporting the use of conventional ANOVA for this comparison.

### 3.5. Telehealth Usability According to Comorbidity

There was a statistically significant difference in mean TUQ scores across the five comorbidity categories. Participants with “other” comorbid conditions had the highest mean TUQ score (118.50 ± 31.71), followed by those with diabetes (89.58 ± 14.87). The lowest mean usability scores were observed among participants with respiratory conditions (83.09 ± 9.44) and chronic kidney disease (84.71 ± 5.97). Participants with hypertension only/no additional comorbidity had a mean TUQ score of 90.06 ± 17.45.

Welch’s ANOVA showed a significant overall difference across comorbidity groups (F(4,113.70) = 14.38, *p* < 0.001; η^2^ ≈ 0.289). Games–Howell comparisons showed that the “other comorbidities” group had significantly higher TUQ scores than hypertension only/no additional comorbidity (adjusted *p* < 0.001), diabetes (adjusted *p* < 0.001), chronic kidney disease (adjusted *p* < 0.001), and respiratory conditions (adjusted *p* < 0.001). No other pairwise comparison was statistically significant after adjustment (Table 6).

### 3.6. Multivariable Predictors of Telehealth Usability

Multiple linear regression was used to examine whether age and clinical characteristics were independently associated with TUQ scores. In the corrected primary model, age was entered as chronological age in years and comorbidity was represented by categorical indicator variables. The model included 264 complete cases. Age was inversely associated with the TUQ score (B = −0.357, standardized β = −0.314, HC3-robust 95% CI −0.525 to −0.190, t = −4.201, *p* < 0.001).

Compared with participants with hypertension only/no additional comorbidity, diabetes was not independently associated with the TUQ score (B = −0.967, β = −0.021, 95% CI −5.898 to 3.965, *p* = 0.700), nor was chronic kidney disease (B = −4.441, β = −0.070, 95% CI −9.746 to 0.865, *p* = 0.101) or respiratory conditions (B = −5.895, β = −0.090, 95% CI −12.559 to 0.769, *p* = 0.083). The “other comorbidities” category was positively associated with the TUQ score (B = 23.292, β = 0.403, 95% CI 12.392 to 34.192, *p* < 0.001).

Sex was not independently associated with the TUQ score (B = 2.221, β = 0.052, 95% CI −2.298 to 6.740, *p* = 0.334). Neither stage II hypertension (B = 0.665, β = 0.015, 95% CI −4.575 to 5.905, *p* = 0.803) nor hypertensive crisis (B = 0.994, β = 0.020, 95% CI −5.319 to 7.306, *p* = 0.757) was independently associated with the TUQ score. Thirty-day readmission was also not statistically significant after adjustment (B = −6.561, β = −0.152, 95% CI −14.362 to 1.240, *p* = 0.099).

The model explained 34.8% of the variance in TUQ score (R^2^ = 0.348; adjusted R^2^ = 0.325). The HC3-robust overall Wald F statistic was 8.53 (*p* < 0.001). VIF values ranged from 1.13 to 1.73, indicating no concerning multicollinearity. Residual diagnostics showed significant departure from normality (Shapiro–Wilk W = 0.938, *p* < 0.001) and heteroscedasticity (Breusch–Pagan *p* < 0.001); therefore, HC3-robust standard errors were used. The maximum Cook’s distance was approximately 0.08, with no highly influential observation identified (Table 7 and Table 8).

## 4. Discussion

Our results indicated that age was associated with perceived telehealth usability, whereas the observed pattern across comorbidity categories was heterogeneous. Participants in the “other comorbidities” group had the highest mean TUQ score, whereas participants with respiratory conditions and chronic kidney disease had some of the lowest mean scores. The multivariable model showed that only the broad “other comorbidities” category differed significantly from hypertension only/no additional comorbidity after adjustment. Diabetes, chronic kidney disease, and respiratory conditions were not independently associated with the TUQ score.

The inverse association between age and telehealth usability in this study is consistent with recent evidence showing that age can influence patients’ experiences with digital health, although age does not predict poorer engagement. In the present study, usability declined across the older age groups, from a mean TUQ score of 110.80 among participants aged 21–30 years to 82.53 among those aged >70 years. However, the post-hoc analysis showed that the between-group pattern was not strictly monotonic. The strongest differences involved the 20–30-year group versus participants aged 41–50, 51–70, and >70 years, with additional differences involving the 31–40-year group. The corrected continuous-age regression nevertheless demonstrated an independent inverse association of age with the TUQ score. A cross-sectional study of 1070 patients with chronic diseases who had used telehealth for at least three months found that older age was associated with lower telehealth satisfaction (coefficient −0.191, *p* < 0.001), while male sex and higher education were associated with greater satisfaction, suggesting that demographic characteristics can materially shape patients’ perceptions of remote care [9]. Similarly, a systematic review and meta-analysis of 19 studies examining technophobia in digital-health contexts found significantly higher technophobia among older adults, with a pooled mean difference of 39.90 (95% CI 35.87–43.94) compared with non-older adults, while digital-health literacy, self-efficacy, social support, and income were among the factors associated with technophobia [14]. This may highlight the greater cognitive, sensory, and procedural demands that digital interfaces can have on older adults, particularly when users should navigate authentication, audiovisual communication, screen interfaces, or data-entry processes. Accordingly, a systematic review and meta-analysis of 59 studies of digital-health acceptance among older adults showed that perceived usefulness was the strongest predictor of behavioral intention (β = 0.72, 95% CI 0.70–0.73), while perceived ease of use was strongly related to perceived usefulness [8]. In a qualitative study of 15 patients participating in a hypertension hybrid-care pathway, patients across three age groups reported improved disease insight, engagement, and shared decision-making, but participants >65 years specifically preferred a simpler telemonitoring application, whereas younger groups preferred more complex information [15]. Moreover, a pilot mixed-methods study of 20 older veterans aged ≥65 years with at least two chronic conditions and at least five medications showed moderate overall engagement and comfort, while technology-related challenges such as audio lag and troubleshooting occurred in more than half of visits but were often successfully overcome with support [16]. The comorbidity findings require cautious interpretation. The descriptive results showed substantial heterogeneity among the specific clinical categories rather than a generalized association of comorbidity with better usability. Participants with hypertension only/no additional comorbidity had a mean TUQ score of 90.06, compared with 89.58 among those with diabetes, 84.71 among those with chronic kidney disease, 83.09 among those with respiratory conditions, and 118.50 among those with other comorbidities. The broad “other” category may encompass clinically diverse conditions and should therefore not be interpreted as evidence that multimorbidity itself improves usability.

A cross-sectional analysis of 3009 adults from a nationally sampled population with hypertension and/or diabetes found that participants with both conditions were older, had lower income and education, and experienced greater social-determinant burden, yet the comorbid group had the highest telehealth utilization and the greatest reliance on phone-based care (21.4%); notably, no significant differences were observed in technical difficulties, perceived quality of care, or privacy concerns across the disease groups [10]. These findings concern utilization rather than multidimensional usability and therefore provide contextual rather than direct evidence for interpreting the present results. Greater healthcare needs may increase the perceived value of remote care in some patients, but this hypothesis cannot be established by the current cross-sectional data. This suggests that a greater burden of chronic disease may increase the perceived value of telehealth sufficiently to offset some of the additional complexity associated with multimorbidity. Patients who require repeated consultations, monitoring, medication adjustments, and coordination across services may have more to gain from remote care than patients with fewer healthcare needs. The positive association between comorbidity and usability should not be interpreted as evidence that all patients with multimorbidity find telehealth easier to use. A cross-sectional usability study involving 38 patients aged >60 years with multimorbidity found a median age of 75 years and a median of eight chronic diseases, demonstrating that multimorbid older adults represent a clinically complex group in which usability needs to be considered alongside cognitive and functional limitations [17].

Sex was not significantly associated with telehealth usability in either the group comparison or multivariable analysis. Men had a slightly higher mean TUQ score than women (94.74 vs. 91.82), but the difference was not statistically significant. This finding differs from the cross-sectional study of 1070 chronic-disease patients, in which male sex was associated with greater telehealth satisfaction, although that study evaluated satisfaction rather than multidimensional usability and included a broader chronic-disease population [9]. The present study therefore provides no evidence of a statistically significant independent association between sex and perceived telehealth usability. It is also possible that differences in education, digital literacy, access to devices, or household support may confound the apparent relationship between sex and telehealth perceptions [18].

Hypertension severity showed a modest unadjusted association with usability, although the conventional ANOVA was close to the prespecified significance threshold (*p* = 0.050) and it was not independently associated with the TUQ score in the corrected multivariable model. Participants experiencing hypertensive crisis had the lowest mean TUQ score. Because the crisis category was based on severe blood-pressure elevation without an acute target-organ-damage criterion, the finding should not be interpreted as an association specifically involving hypertensive emergency. Recent evidence indicates that telehealth can remain useful across different levels of hypertension severity when paired with active monitoring and treatment adjustment [19]. A meta-analysis of 14 randomized controlled trials found that mobile-health interventions improved systolic and diastolic blood pressure, blood-pressure control, treatment adherence, hypertension knowledge, and quality of life, with effects generally consistent across age groups and intervention modalities [20].

Policymakers should avoid assuming that a single telehealth interface is suitable for all patients and should instead require age-sensitive design features, including simplified navigation, larger and clearer visual elements, straightforward login processes, accessible language, and alternative communication channels for patients who experience difficulty with video-based systems [21]. These features should be considered implementation options rather than direct effects demonstrated by the current study. For healthcare policymakers in Saudi Arabia, a flexible telehealth model may be appropriate in which digital care can be supplemented by alternative communication channels, caregiver-supported use, technical assistance, and clinician-led escalation when digital interaction is insufficient.

There are several limitations that require consideration. First, the cross-sectional analysis prevents determination of temporal or causal relationships between age, comorbidity, and usability perceptions. Second, participants were selected using purposive sampling from two hospitals in Sakaka, Saudi Arabia, which may limit generalizability to other Saudi healthcare settings and to patients with hypertension who do not use telehealth. In particular, individuals who were unable or unwilling to engage with telehealth were not represented and may have different or greater usability barriers. Third, the TUQ was self-reported and therefore susceptible to recall, reporting, and social-desirability biases. Fourth, important determinants of digital-health experience such as digital literacy and health literacy were not incorporated into the model. Fifth, the specific telehealth modality, number of telehealth contacts, and duration of encounters were not separately captured, limiting the ability to determine whether usability differed by type or intensity of telehealth exposure. Sixth, the individual TUQ domain scales demonstrated modest internal consistency in the present sample (α = 0.342–0.486), although total TUQ reliability was stronger (α = 0.866). Consequently, domain findings were treated primarily as descriptive and the total TUQ score was used as the primary inferential outcome. Finally, the broad “other comorbidities” category may obscure clinically important differences between individual conditions, and the single-category comorbidity variable does not capture the number, severity, duration, or combinations of coexisting diseases.

## 5. Conclusions

This study demonstrates that perceived telehealth usability among patients with hypertension is not uniform across the population and is associated with age and varies across specific clinical subgroups. In the corrected multivariable analysis, each additional year of age was associated with a 0.36-point lower TUQ score, whereas only the broad “other comorbidities” category showed a statistically significant positive adjusted association compared with hypertension only/no additional comorbidity. Diabetes, chronic kidney disease, and respiratory conditions were not independently associated with the TUQ score. These findings highlight the importance of designing accessible, patient-centered telehealth services that account for differences in age, clinical complexity, and individual support needs. Future research should use larger, multicenter prospective studies to examine how age-related factors, multimorbidity, digital and health literacy, socioeconomic circumstances, caregiver support, previous technology experience, and telehealth modality and intensity of use shape telehealth usability and sustained engagement.

## Figures and Tables

**Table 1:** Sociodemographic and clinical characteristics of the study population.

Characteristic	*n*	%
Age group (years) *		
<20	5	1.9
20–30	44	16.6
31–40	56	21.1
41–50	57	21.5
51–70	73	27.5
>70	30	11.3
Sex		
Male	117	44.0
Female	149	56.0
Hypertension severity		
Stage I	105	39.5
Stage II	98	36.8
Hypertensive crisis	63	23.7
Comorbidity status		
Hypertension only/no additional comorbidity	79	29.7
Diabetes	77	28.9
Chronic kidney disease	34	12.8
Respiratory conditions	32	12.0
Other comorbidities	44	16.5
30-day readmission		
Yes	152	57.1
No	113	42.5
Missing	1	0.4

* Age percentages are based on the 265 participants with valid age data.

**Table 2:** Mean TUQ scores and internal consistency.

TUQ Domain	Total Score, Mean ± SD	Mean Item Score, Mean ± SD	Cronbach’s α
Usefulness	12.95 ± 3.96	4.32 ± 1.32	0.406
Ease of use and learnability	17.65 ± 4.74	4.41 ± 1.19	0.384
Interface quality	13.06 ± 4.31	4.35 ± 1.44	0.486
Interaction quality	17.94 ± 4.68	4.48 ± 1.17	0.368
Reliability	17.89 ± 4.56	4.47 ± 1.14	0.388
Satisfaction and future use	13.61 ± 3.74	4.54 ± 1.25	0.342
Overall TUQ	93.11 ± 21.58	4.43 ± 1.03	0.866

Note: The domain structure was 3, 4, 3, 4, 4, and 3 items for usefulness, ease of use and learnability, interface quality, interaction quality, reliability, and satisfaction/future use, respectively.

**Table 3:** Mean TUQ scores according to age group.

Age Group, Years	*n*	Mean TUQ ± SD
<20	5	88.40 ± 4.28
20–30	44	110.75 ± 31.72
31–40	56	97.29 ± 24.56
41–50	57	86.58 ± 10.36
51–70	73	88.97 ± 15.58
>70	30	82.53 ± 7.33

**Table 4:** Normality and homogeneity-of-variance diagnostics.

Comparison	Groups with Significant Shapiro–Wilk Test	Levene’s W (*p*)	Primary Omnibus Test
Age group	20–30, 31–40, 41–50, 51–70	13.71 (<0.001)	Welch F(5,46.07) = 8.96, *p* < 0.001
Comorbidity group	Hypertension only, diabetes, respiratory conditions, other comorbidities	17.68 (<0.001)	Welch F(4,113.70) = 14.38, *p* < 0.001

**Table 5:** Games–Howell pairwise comparisons for age groups.

Comparison	Adjusted *p* Value	Hedges’ g
<20 vs. 20–30	0.001	−0.72
<20 vs. 31–40	0.204	−0.37
<20 vs. 41–50	0.966	0.18
<20 vs. 51–70	1.000	−0.04
<20 vs. >70	0.219	0.81
20–30 vs. 31–40	0.198	0.48
20–30 vs. 41–50	<0.001	1.08
20–30 vs. 51–70	0.001	0.94
20–30 vs. >70	<0.001	1.12
31–40 vs. 41–50	0.040	0.57
31–40 vs. 51–70	0.242	0.41
31–40 vs. >70	0.001	0.72
41–50 vs. 51–70	0.900	−0.18
41–50 vs. >70	0.293	0.42
51–70 vs. >70	0.058	0.47

**Table 6:** Games–Howell pairwise comparisons for comorbidity groups.

Comparison	Adjusted *p* Value	Hedges’ g
Hypertension only vs. diabetes	1.000	0.03
Hypertension only vs. chronic kidney disease	0.118	0.36
Hypertension only vs. respiratory conditions	0.060	0.44
Hypertension only vs. other comorbidities	<0.001	−1.20
Diabetes vs. chronic kidney disease	0.106	0.38
Diabetes vs. respiratory conditions	0.058	0.48
Diabetes vs. other comorbidities	<0.001	−1.28
Chronic kidney disease vs. respiratory conditions	0.922	0.20
Chronic kidney disease vs. other comorbidities	<0.001	−1.38
Respiratory conditions vs. other comorbidities	<0.001	−1.41

**Table 7:** Sensitivity analysis using categorical age group.

Predictor	B	HC3 95% CI	*p*
<20 vs. 20–30	−11.546	−21.476 to −1.615	0.023
31–40 vs. 20–30	−6.280	−16.900 to 4.340	0.245
41–50 vs. 20–30	−16.024	−25.804 to −6.243	0.001
51–70 vs. 20–30	−15.023	−25.191 to −4.855	0.004
>70 vs. 20–30	−20.876	−32.616 to −9.135	<0.001

**Table 8:** Corrected multivariable linear regression model for TUQ score.

Predictor	B	Standardized β	HC3 95% CI	t	*p*
Age, per year	**−0.357**	**−0.314**	**−0.525 to −0.190**	**−4.201**	**<0.001**
Female vs. male	2.221	0.052	−2.298 to 6.740	0.968	0.334
Stage II vs. Stage I	0.665	0.015	−4.575 to 5.905	0.250	0.803
Hypertensive crisis vs. Stage I	0.994	0.020	−5.319 to 7.306	0.310	0.757
Diabetes vs. hypertension only/no additional comorbidity	−0.967	−0.021	−5.898 to 3.965	−0.386	0.700
Chronic kidney disease vs. hypertension only/no additional comorbidity	−4.441	−0.070	−9.746 to 0.865	−1.648	0.101
Respiratory conditions vs. hypertension only/no additional comorbidity	−5.895	−0.090	−12.559 to 0.769	−1.742	0.083
Other comorbidities vs. hypertension only/no additional comorbidity	**23.292**	**0.403**	**12.392 to 34.192**	**4.208**	**<0.001**
30-day readmission: yes vs. no	−6.561	−0.152	−14.362 to 1.240	−1.656	0.099

Model R^2^ = 0.348; adjusted R^2^ = 0.325; HC3-robust Wald F(9,254) = 8.53, *p* < 0.001.

## Data Availability

The data that support the findings of this study are available on request from the corresponding author. The data are not publicly available due to privacy or ethical restrictions.

## Abbreviations

ANOVA	Analysis of variance
ASU	Applied Science Private University
CI	Confidence interval
CNS	Clinical Nurse Specialist
EHR	Electronic health record
MA	Master of Arts
MBRU	Mohammed Bin Rashid University of Medicine and Health Sciences
PhD	Doctor of Philosophy
RN	Registered Nurse
SD	Standard deviation
SPSS	Statistical Package for the Social Sciences
SSMC	Sheikh Shakhbout Medical City
TUQ	Telehealth Usability Questionnaire
SBP	Systolic blood pressure
DBP	Diastolic blood pressure
UAE	United Arab Emirates
